# Neuroprotective effects of dexmedetomidine against hyperoxia-induced injury in the developing rat brain

**DOI:** 10.1371/journal.pone.0171498

**Published:** 2017-02-03

**Authors:** Stefanie Endesfelder, Hanan Makki, Clarissa von Haefen, Claudia D. Spies, Christoph Bührer, Marco Sifringer

**Affiliations:** 1 Department of Neonatology, Charité – Universitätsmedizin Berlin, Berlin, Germany; 2 Department of Anesthesiology and Intensive Care Medicine, Charité – Universitätsmedizin Berlin, Berlin, Germany; Universitatsklinikum Wurzburg, GERMANY

## Abstract

Dexmedetomidine (DEX) is a highly selective agonist of α2-receptors with sedative, anxiolytic, and analgesic properties. Neuroprotective effects of dexmedetomidine have been reported in various brain injury models. In the present study, we investigated the effects of dexmedetomidine on hippocampal neurogenesis, specifically the proliferation capacity and maturation of neurons and neuronal plasticity following the induction of hyperoxia in neonatal rats. Six-day old sex-matched Wistar rats were exposed to 80% oxygen or room air for 24 h and treated with 1, 5 or 10 μg/kg of dexmedetomidine or normal saline. A single pretreatment with DEX attenuated the hyperoxia-induced injury in terms of neurogenesis and plasticity. In detail, both the proliferation capacity (PCNA+ cells) as well as the expression of neuronal markers (Nestin+, PSA-NCAM+, NeuN+ cells) and transcription factors (SOX2, Tbr1/2, Prox1) were significantly reduced under hyperoxia compared to control. Furthermore, regulators of neuronal plasticity (Nrp1, Nrg1, Syp, and Sema3a/f) were also drastically decreased. A single administration of dexmedetomidine prior to oxygen exposure resulted in a significant up-regulation of expression-profiles compared to hyperoxia. Our results suggest that dexmedetomidine may have neuroprotective effects in an acute hyperoxic model of the neonatal rat.

## Introduction

Improving advances in neonatal intensive care survival rates of preterm children continues, but extremely preterm children still have high rates of morbidity [1,2]. External factors such as oxidative stress, intensified by additional mechanical ventilation, and the need for the administration of medications or surgical interventions lead to additional burdens for the immature organism. Oxidative stress promotes the development and pathogenesis of complications in premature infants [3–9], because the antioxidant defense system is poorly developed [10].

The inadvertent oxygen oversupply secondary to change from the intra- to extra-uterine environment is aggravated by additional oxygen supplementation during neonatal intensive care. This triggers the formation of reactive oxygen radicals that contribute to oxidative changes in proteins, lipids and nucleic acids [11] and negatively impacts on survival of neuronal cells during development [12]. Hyperoxia leads to an increased expression of pro-inflammatory cytokines in the immature rat brain [13,14], which is associated with neuronal degradation [15]. Further studies show that hyperoxic conditions promote the risk for neurobehavioral cognitive delayed effects as well as the development of cerebral palsy in preterm infants [16,17]. The use of anesthetics and sedatives for intensive medical treatments in preterm infants is often imperative, therefore the correct balance between good oxygen saturation and the use of potentially neurodegenerative sedatives is an important aspect [18,19]. Furthermore, the use of narcotics and sedatives in neonatology leads to impairments of the developing brain and thus to a higher incidence of intraventricular hemorrhage (IVH) and periventricular leukomalacia (PVL) [19,20]. Brain injury associated with IVH, PVL, seizures, or sepsis, can trigger cognitive developmental delays, motor impairments and behavioral disorders [21]. Neurogenesis, synaptogenesis, and connectivity are important processes that occur in the developing brain, mainly in the hippocampus for learning and memory, during the neonatal period in humans and rodents [22]. It is known that the hippocampal structures are particularly vulnerable to many stressors and medical complications, such as infection and oxidative stress [15,23–25]. Consequences include reduction in grey matter [25], impairment of neuronal migration and plasticity [15,26,27], and neuronal damage [28].

The highly selective α2 agonist dexmedetomidine (DEX) exerts its effect through sympatholysis and displays sedative, analgesic, and anxiolytic properties, but also side effects such as hypotension or bradycardia [29,30]. DEX has positive effects in comparison to other sedatives, including reduction in respiratory depression and hypotension, delirium diminution, decrease of lung and kidney damage, and reduction of neural apoptosis [31–33]. A medication strategy with DEX for preterm infants provides effective sedation, shorter ventilation duration, and a reduction in the incidence of sepsis [34–36]. Taking into account the damaging effects of oxidative stress, the possible neuroprotection afforded by DEX as a sedative in pediatrics warrants further investigation [33,37–42].

To date, little is known about the impact of dexmedetomidine on the developing brain. Therefore, this study aims to investigate the effect of dexmedetomidine on neurogenesis in the dentate gyrus in terms of proliferation capacity, neuronal maturation, and neuronal plasticity in a hyperoxia-mediated brain injury model of the neonatal rat.

## Materials and methods

### Animals and drug administration

All procedures were approved by the state animal welfare authorities (LAGeSo G-0145/13) and followed institutional guidelines. Six-day old Wistar rats from time-pregnant dams were obtained from Charité-Universitätsmedizin Berlin (Germany) and randomly assigned to cages and treatment.

The animal experiments were carried out as previously described [33]. Dexmedetomidine (DEX; dexdor^®^, Orion Pharma, Espoo, Finland) was dissolved in phosphate buffered saline. Three doses of the drug (1, 5, and 10 μg/kg body weight) were used and all injections were given intraperitoneally (i.p.) as a fixed proportion of body weight (100 μl/10 g). The rat pups were divided into different experimental groups (description with the relevant experimental abbreviations): (1) control group (CON; 21% O_2_, room air) with 0,9% saline, (2) verum group (21% O_2_) with 1 μg/kg DEX (DEX1), (3) verum group with 5 μg/kg DEX (DEX5), (4) verum group with 10 μg/kg DEX (DEX10), (5) hyperoxia group (HY; 80% O_2,_ OxyCycler BioSpherix, Lacona, NY, USA) with 0,9% saline, (6) hyperoxia with 1 μg/kg DEX (HYDEX1), (7) hyperoxia with 5 μg/kg DEX (HYDEX5), and (8) hyperoxia with 10 μg/kg DEX (HYDEX10). Of 10 rat pups in each group with different gender, 5 were used for gene expression analysis (quantitative realtime PCR and Western blot) and 5 for immunohistological assessments (in each subgroup 2 males/ 3 females or 3 males/ 2 females). For hyperoxia or normoxia exposure, pups were kept together with their dams. Saline or DEX were administrated once 15 min before the start of oxygen exposure.

### Tissue preparation

After 24 h (P7) of exposure, rats were transcardially perfused with phosphate buffered saline (PBS, pH 7.4) under anesthesia (i.p.) with ketamine (50 mg/kg), xylazine (10 mg/kg), and acepromazine (2 mg/kg) then decapitated. The olfactory bulb and cerebellum were removed, brain hemispheres were snap-frozen in liquid nitrogen, and stored at -80°C. For immunohistochemical analysis, animals were perfused with PBS followed by perfusion with 4% paraformaldehyde at pH 7.4, the brains were postfixed at 4°C for 1 day, embedded in paraffin, and processed for histological staining.

### Tissue fixation

The sections (5 μm) of paraffin-embedded brains were mounted onto Super Frost plus-coated slides (R. Langenbrinck, Emmendingen, Germany) and were deparaffinized in Roti-Histol (Carl Roth, Karlsruhe, Germany) twice for 10 min each, then rehydrated in ethanol (100, 90, 80, and 70%), distilled water, and PBS for 3 min each at room temperature.

### Immunostaining of neuronal and proliferation markers

Immunostaining was performed as previously described [33]. Briefly, sections were fixed in citrate buffer (pH 6.0) at 600 W for 12 min in a microwave oven to increase cell membrane permeability and thus, demasking intracellular epitopes. Afterward, sections were cooled and washed three times with PBS. For the primary antibody PSA-NCAM, sections were additionally incubated for 90 min in 50% formamide/ 2xSSC buffer (3 M sodium chloride, 300 mM sodium citrate) at 65°C. After two five minute washes in 2xSSC buffer for 30 min, the sections were incubated at 37°C in 2 M hydrochloric acid then 10 min in 0.1 M borate buffer at room temperature. Finally, the sections were washed four times for 5 min in PBS.

The slices were blocked with blocking buffer (10% goat serum, 1% BSA, and 0.3% Triton X-100 in PBS) for 2 h at room temperature. Sections were washed once with PBS and subsequently incubated overnight at 4°C with either monoclonal mouse anti-rat nestin (1:200, Millipore, Darmstadt, Germany), monoclonal mouse anti-rat NeuN (1:500, Millipore), or monoclonal mouse anti-rat PSA-NCAM (1:200, Millipore) diluted in antibody diluent for reducing background (DAKO Deutschland GmbH, Hamburg, Germany). Slices were washed three times in PBS. The secondary Cy3-conjugated goat anti-mouse IgG (Millipore) was applied at a dilution of 1:200 in the carrier solution of the primary antibody and incubated at room temperature in the dark overnight. For double staining with the proliferation marker, proliferating cell nuclear antigen (PCNA), the sections were washed once with PBS and incubated with blocking buffer for 60 min in the dark. After washing with PBS, sections were incubated with rabbit anti-rat PCNA (1:50, Abcam, Cambridge, UK) in antibody diluent (DAKO) overnight at 4°C. The secondary labeling with AlexaFluor 488-conjugated goat anti-rabbit IgG (Invitrogen, Carlsbad, CA, USA) including 4,6-diamidino-2-phenylindole (DAPI, 10 ng/ml) was incubated for 60 min in the dark. After three washes with PBS, slides were mounted with mounting media (Vectashield HardSet Mounting Media, Vector Laboratories, Burlingame, CA, USA).

Sections of the hippocampus were viewed blinded under fluorescent light using a Keyence BZ-9000 microscope (BIOREVO) equipped with a 200 x magnification objective and analyzed with BZII-analyzer software (KEYENCE Deutschland GmbH, Neu-Isenburg, Germany). Images for PCNA-labeled and nestin, PSA-NCAM, and NeuN/ PCNA double-labeled cells within the granule cell layer (GCL, dentate gyrus) and the polymorphic layer (PL, hilus) including the subgranular zone were counted in 4 separate sections per animal. We analyzed labeled cell numbers using Adobe Photoshop C3 10.0 (Adobe Systems Incorporated, San Jose, CA, USA).

### RNA extraction and quantitative real-time PCR

The gene expression analysis was performed as previously described [15]. Total RNA was isolated from snap-frozen tissue by acidic phenol/chloroform extraction (peqGOLD RNAPure^™^; PEQLAB Biotechnologie, Erlangen, Germany) and 2 μg of RNA was reverse transcribed. The PCR products of *hypoxanthine-guanine phosphoribosyl-transferase* (*HPRT*), *neuregulin 1* (*Nrg1*), *neuropilin 1* (*Nrp1*), *paired box 6* (*Pax6*), *prospero homeobox 1* (*Prox1*), *semaphorin 3A* (*Sema3a*), *semaphorin 3F* (*Sema3f*), *sex determining region Y-box 2* (*SOX2*), *synaptophysin* (*Syp*), *T-box brain gene 1* (*Tbr1*), and *T-box brain gene 2* (*Tbr2*) were quantified in real time, using dye-labeled fluorogenic reporter oligonucleotide probes with the sequences summarized in Table 1. All probes were labeled at their 5´ ends with the reporter dye 6-carboxy-fluoresceine (FAM) and at their 3´ ends with the quencher dye 6-carboxy-tetramethylrhodamine (TAMRA). PCR and detection were performed in triplicate and repeated two times for each sample in 11 μl reaction mix, which contained 5 μl of 2× KAPA PROBE FAST qPCR Mastermix (PEQLAB Biotechnologie), 2.5 μl of 1.25 μM oligonucleotide mix, 0.5 μl (0.5 μM) of probe (BioTeZ, Berlin, Germany), and 3 to 25 ng of cDNA templates with *HPRT* used as an internal reference. The PCR amplification was performed in 96-well optical reaction plates for 40 cycles with each cycle at 94°C for 15 s and 60°C for 1 min. The expression of target genes was analyzed with the StepOnePlus real-time PCR system (Applied Biosystems, Life Technologies, Carlsbad, CA, USA) according to the 2^−ΔΔ*C*T^ method [43].

**Table 1:** Sequences of oligonucleotides and gene locus.

Gene		Oligonucleotide sequences 5´- 3´	
***HPRT***	forward	GGAAAGAACGTCTTGATTGTTGAA	NM_012583.2
	reverse	CCAACACTTCGAGAGGTCCTTTT	
	probe	CTTTCCTTGGTCAAGCAGTACAGCCCC	
***Nrg1***	forward	GGGACCAGCCATCTCATAAA	NM_001271118
	reverse	ATCTTGACGGGTTTGACAGG	
	probe	ACTTTCTGTGTGAATGGGGG	
***Nrp1***	forward	TGAGCCCTGTGGTCTATTCC	NM_145098
	reverse	CCTCTGGCTTCTGGTAGTGC	
	probe	TGTGGGTACACTGAGGGTCA	
***Pax6***	forward	TCCCTATCAGCAGCAGTTTCAGT	NM_013001.2
	reverse	GTCTGTGCGGCCCAACAT	
	probe	CTCCTCCTTTACATCGGGTT	
***Prox1***	forward	TGCCTTTTCCAGGAGCAACTAT	NM_001107201
	reverse	CCGCTGGCTTGGAAACTG	
	probe	ACATGAACAAAAACGGTGGC	
***Sema3a***	forward	GAAAACGGTCGTGGGAAGAG	NM_017310
	reverse	AGCAAAGTCTCGTCCCATGA	
	probe	GACCCCAAACTTCTGACTGC	
***Sema3f***	forward	CCATGCGCACAGATCAGTAC	NM_001108185
	reverse	AGTTTATCGTCGTTGCGCTC	
	probe	CGGTGGCTCAATGATCCTTC	
***SOX2***	forward	ACAGATGCAGCCGATGCA	NM_001109181
	reverse	GGTGCCCTGCTGCGAGTA	
	probe	CAGTACAACTCCATGACCAG	
***Syp***	forward	TTCAGGCTGCACCAAGTGTA	NM_012664
	reverse	TTCAGCCGACGAGGAGTAGT	
	probe	AGGGGGCACTACCAAGATCT	
***Tbr1***	forward	TCCCAATCACTGGAGGTTTCA	NM_0011911070
	reverse	GGATGCATATAGACCCGGTTTC	
	probe	AAATGGGTTCCTTGTGGCAA	
***Tbr2***	forward	ACGCAGATGATAGTGTTGCAGTCT	XM_001061749.2
	reverse	ATTCAAGTCCTCCACACCATCCT	
	probe	CACAAATACCAACCTCGACT	

***HPRT*:** hypoxanthine-guanine phosphoribosyl-transferase; ***Nrg1*:** neuregulin 1; ***Nrp1*:** neuropilin 1; ***Pax6*:** paired box 6; ***Prox1*:** prospero homeobox 1; ***Sema3a*:** semaphorin 3A; ***Sema3f*:** semaphorin 3F; ***SOX2*:** sex determining region Y-box 2; ***Syp*:** synaptophysin; ***Tbr1/2*:** T-box brain gene 1/2.

### Protein extraction

Protein was extracted as previously described [15,33]. Briefly, snap-frozen brain tissue was homogenized in RIPA buffer solution for protein extraction. The homogenate was centrifuged at 3000 g (4°C) for 10 min, the microsomal fraction was subsequently centrifuged at 17000 g (4°C) for 20 min, and stored at -80°C until further analysis. After collecting the supernatant, protein concentrations were determined using the Pierce BCA kit (Pierce/Thermo Scientific, Rockford, IL, USA) with 30 min incubation at 37°C prior to spectrophotometry at 562 nm.

### Immunoblotting

Western blotting was performed as previously described [15,33]. Briefly, protein extracts (25–50 μg per sample) were denaturated in Laemmli sample loading buffer at 95°C, size-fractionated by 8–10% sodium dodecyl sulfate polyacrylamide gel electrophoresis, and electrotransferred in transfer buffer to a nitrocellulose membrane (0.2 μm pore, Bio-Rad, Munich, Germany). Nonspecific protein binding was prevented by treating the membrane with 5% nonfat dry milk in Tris-buffered saline /0.1% Tween 20 for 1 h at room temperature. Equal loading and transfer of proteins were confirmed by staining the membranes with Ponceau S solution (Fluka, Buchs, Switzerland). The membranes were incubated overnight at 4°C with mouse monoclonal anti-NRG1 (44 kDa; 1:500; Santa Cruz, Heidelberg, Germany), goat polyclonal anti-NRP1 (120 kDa; 1:1.000; R&D Systems, Minneapolis, MN, USA), rabbit polyclonal anti-SEMA3A (89 kDa; 1:1.000; Abcam, Cambridge, UK), rabbit polyclonal anti-SEMA3F (88 kDa; 1:1.000; Abcam) or rabbit monoclonal anti-SYP (34 kDa; 1:1.000; Abcam), respectively. Secondary incubations were performed with horseradish peroxidase-linked anti-mouse (1:2000; Dako, Glostrup, Denmark), anti-goat (1:2000; Vector Laboratories) or anti-rabbit (1:2000; Dako) antibody. Positive signals were visualized using the SuperSignal^™^ West Pico kit (Pierce) according to manufacturer´s directions and quantified using a ChemiDoc^™^ XRS+ system and Image Lab^™^ software (Bio-Rad). Membranes were stripped, washed, blocked, and re-probed overnight at 4°C with mouse anti-β-actin monoclonal antibody (42 kDa; 1:10.000; Sigma-Aldrich, Munich, Germany). Each experiment was repeated three times.

### Statistical analyses

All data are expressed as the mean ± standard error of the mean (SEM). Groups were compared using a one-way analysis of variance (ANOVA), and significance was determined using Bonferroni's correction for multiple comparisons with independent sample *t* test. A two-sided *p* value of <0.05 was considered significant. All graphics and statistical analyses were performed using the GraphPad Prism 5.0 software (GraphPad Software, La Jolla, CA, USA).

## Results

### Neuronal differentiation and proliferation

The dentate gyrus is one of the brain structures where neurogenesis occurs during fetal brain development and in the adult brain (Deng et al., 2010). The proliferation capacity of neural cells is a critical factor for physiological development, and numerous environmental agents and physiological mediators can modify neural proliferation (reviewed in [44,45]).

**Fig 1:**
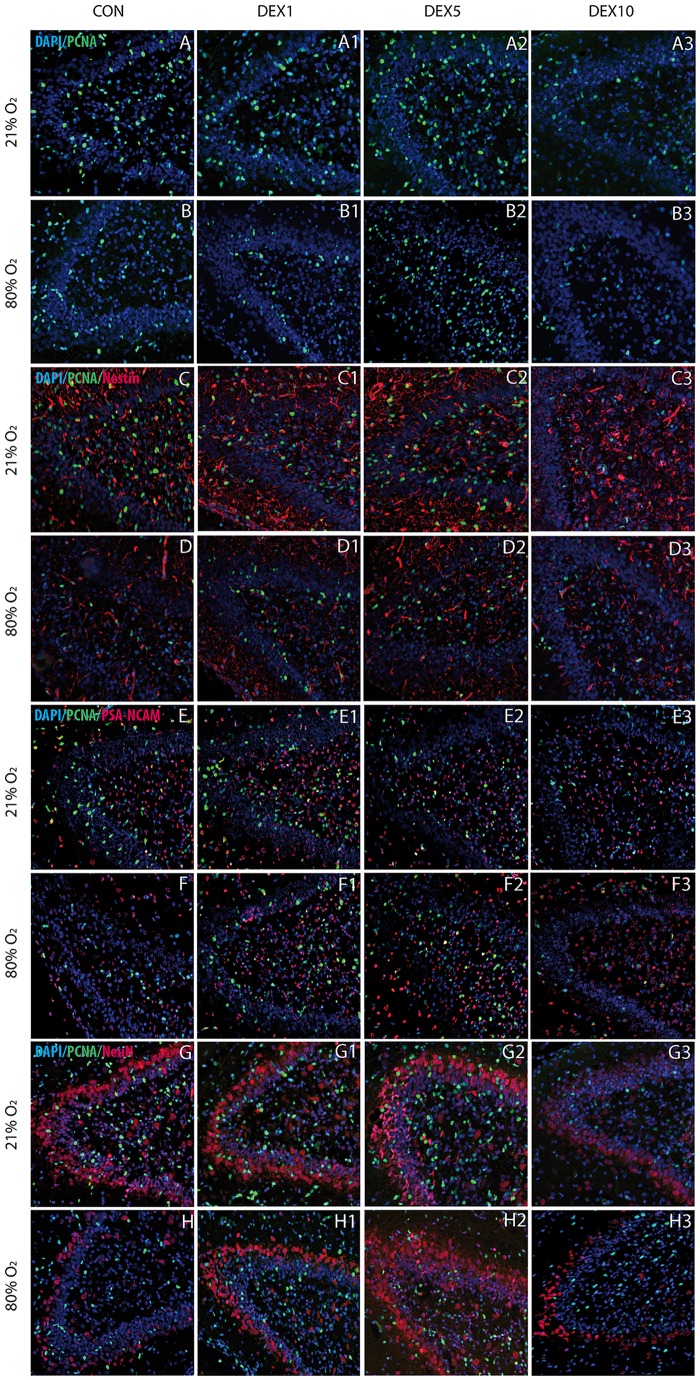
Paraffin sections of the hippocampus at postnatal day (P)7 of Wistar rats stained with A/B) proliferating cell nuclear antigen (PCNA)/DAPI; double immunofluorescence staining with C/D) Nestin/PCNA/DAPI; E/F) polysialylated neuronal cell adhesion molecule (PSA-NCAM)/PCNA/DAPI; G/H) neuronal nuclei (NeuN)/PCNA/DAPI. Hyperoxia in neonatal rats decreased the proliferation positive cells (PCNA+, green, **A/B**) and the expression of neuronal marker for neuronal progenitor cells (Nestin+, red, **C/D**), immature neurons (PSA-NCAM+, red, **E/F**), and mature postmitotic neurons (NeuN+, red, **G/H**). Application of DEX1 and/or DEX5 under hyperoxic exposure resulted in improved expression of neuronal markers and increase of proliferation in granular cell layer and polymorphic layer of DG. DEX10 led to a reduction of cell counts of NeuN in hyperoxic animals. Under normoxic conditions DEX1 and/or DEX5 upregulated PCNA and the differentiation marker and DEX10 showed negative effects on PNCA and NeuN expression. All images were taken at identical magnification (original magnification 200 x).

**Fig 2:**
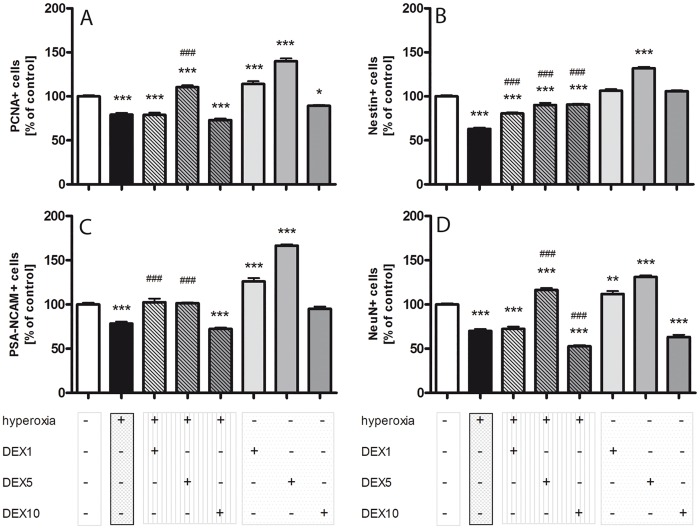
Quantitation of A) PCNA+, B) Nestin+, C) PSA-NCAM+, and D) NeuN+ cell counts in sum of the granular cell layer and polymorphic layer with DEX (1, 5, and 10 μg/kg) under hyperoxia (hatched grey bars), hyperoxia alone (black bars), DEX under normoxic conditions (plain grey bars), and in comparison to normoxia control group (100%, white bars). Data are expressed relative to the normoxia-exposed control group as mean ± SEM of n = 5. The 100% values are for PCNA 258.9, for Nestin 354.1, for PSA-NCAM 209.3, and for NeuN 333.3. * p<0.05, ** p<0.01, and *** p<0.001 *versus* control; ^###^ p<0.001 *versus* hyperoxia (t-test, Bonferroni post hoc test after one-way ANOVA).

To investigate the impact of DEX at different concentrations (1 μg/kg DEX1; 5 μg/kg DEX5; 10 μg/kg DEX10) on the effects caused by oxygen toxicity, proliferation capacity was analyzed by the proliferation marker PCNA (Fig 1A and 1B), an auxiliary protein of DNA polymerase δ which peaks at the G1/S interface of the cell cycle [46]. We analyzed PCNA positive cells in sum of granule cell layer and polymorphic layer (Fig 2A) and observed a drastic reduction of proliferating cells after hyperoxia exposure compared to normoxia control (Fig 1A and 1B). The application of DEX1 (Fig 1A1) and DEX5 (Fig 1A2) under normoxic conditions showed a significant increase in PCNA positive cell counts and a reduction by DEX10 (Fig 1A3). DEX5 significantly improved the reduced proliferation rate under hyperoxia (Fig 1B2).

### Dexmedetomidine improves the hyperoxia-induced delayed maturation

Hippocampal neurogenesis occurs in the dentate gyrus through the emergence of new neurons from neural progenitor cells. The formation of new dentate granule neurons is a multifaceted-regulated process [47] and the steps of differentiation and maturation are characterized by specific neuronal markers [47,48].

A neural progenitor marker expressed during development of CNS is nestin, a class VI intermediate filament protein [49]. Hyperoxic conditions led to a significant reduction in nestin-positive cell counts in the dentate gyrus (Fig 2B) and nestin expression (Fig 1C and 1D). DEX1 (Fig 1D1), DEX5 (Fig 1D2), and DEX10 (Fig 1D3) enhanced nestin expression under hyperoxic conditions and DEX5 treatment under normoxia showed an increase (Fig 1C1, 1C2 and 1C3).

A neuronal marker associated with differentiation and migration is polysialylated neuronal cell adhesion molecule (PSA-NCAM), expressed in mitotically-maturing neurons [50]. PSA-NCAM expression after 24 hours exposure to 80% oxygen was reduced (Figs 1E, 1F and 2C). The application of DEX1 and DEX5 increased the cell number of PSA-NCAM-expressing cells in the DG (Figs 1F1 and 2) under hyperoxic exposure. An enhanced PSA-NCAM cell count was also confirmed under normoxic conditions for DEX1 (Fig 1E1) and DEX5 (Fig 1E2).

An excellent marker for postmitotic mature neurons is the neuron-specific nuclear protein neuronal nuclei (NeuN) [51]. The number of NeuN-positive mature neurons (Fig 1G and 1H) and NeuN cell counts (Fig 2D) were significantly reduced by hyperoxia. At this stage of neuronal maturation DEX5 showed protective effects (Fig 1H2). DEX10 enhanced the hyperoxia-reduced differentiation (Fig 1H3). Under normoxic conditions DEX1 (Fig 1G1) and DEX5 (Fig 1G2) increased the number of the NeuN-positive cells. The inhibitory effects of DEX10 could be detected even under normoxia (Fig 1G3).

### Dexmedetomidine modulates neuronal transcriptional network

Hippocampal neurogenesis in the developing brain is a multistep-regulated process with sequential expressed transcription factors (TF). The transcriptional network is arranged in series that overlap and specific TF can be used to identify differentiation stages. An early TF of proliferative neural progenitors is Pax6, a paired domain and homeodomain-containing transcription factor. SOX2, a member of the SOXB1 subgroup (SOX 1–3) that is strongly expressed in dividing cells and is located at the junction of the precursor cells to immature neurons. The expression overlaps with up-regulation of Tbr2, a T-box transcription factor and a TF of mitotically intermediate neurons. Postmitotic neurons are characterized by the expression of Tbr1 and Prox1, a homeobox transcription factor homologous to the *Drosophila melanogaster* gene prospero, expressed in mature neurons (reviewed in [52]).

**Fig 3:**
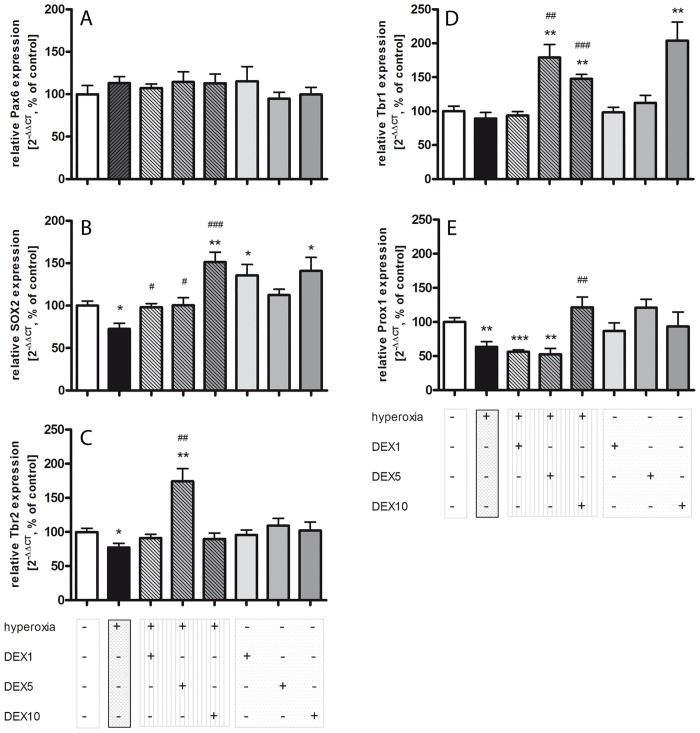
Expression of mediators of transcriptional network is decreased in neonatal rats after hyperoxic injury and upregulated with DEX. The relative mRNA expressions of transcription factors were measured in rat brain homogenates with DEX (1, 5, and 10 μg/kg) under hyperoxia (hatched grey bars), hyperoxia alone (black bars), DEX under normoxic conditions (plain grey bars), and in comparison to normoxia control group (100%, white bars) by quantitative realtime PCR. **A)** There are no changes under normoxia/hyperoxia with or without DEX for Pax6. **B)** Note the significant reduction of SOX2 under hyperoxia and the increase with a single dose of DEX. Under normoxic conditions with DEX1 and DEX10 the mRNA expression of SOX2 was increased significantly. **C)** Tbr2 mRNA expression was reduced under hyperoxia and upregulated at DEX5. **D)** Hyperoxia has no influence on the mRNA expression of Tbr1, but DEX5 and DEX10 triggered the expression above the normoxia level. The same effect was detected for DEX10 under normoxia. **E)** There was a significant decrease for Prox1 mRNA expression under hyperoxia alone and with DEX1 and DEX5, but also a significant up-regulation with DEX10. Data shown as mean ± SEM, n = 5 per group. * p<0.05, ** p<0.01, and *** p<0.001 *versus* control; ^##^ p<0.01 and ^###^ p<0.001 *versus* hyperoxia (t-test, Bonferroni post hoc test after one-way ANOVA).

The early TF Pax6 is not affected by high concentrations of oxygen or by different DEX concentrations (Fig 3A). Hyperoxia reduced the expression of SOX2 (Fig 3B). DEX1 and DEX5 improved this effect on normoxic level, and DEX10 significantly enhanced the expression of SOX2 beyond. Under normoxic conditions both DEX1 and DEX10 led to an increased expression of SOX2. The TF Tbr2 expression was also reduced by exposure to hyperoxia (Fig 3C) and here, only DEX5 significantly reversed this reduction. Under normoxia, there were no changes in Tbr2 expression by DEX. No change in Tbr1 expression was observed under hyperoxia, but DEX5 (Fig 3D) and DEX10 still led to a significant increase. Dex10 induced this increase even under normoxic conditions. Interestingly, Prox1 was changed by both, hyperoxia alone (Fig 3E) but DEX1 and DEX5 showed no improving effect on Prox1 expression. DEX10 solely abolished this reduction. No changes were detected with DEX treatment during normoxia. Gender-dependent differences were not determined.

### Dexmedetomidine improves neuronal plasticity under hyperoxia

**Fig 4:**
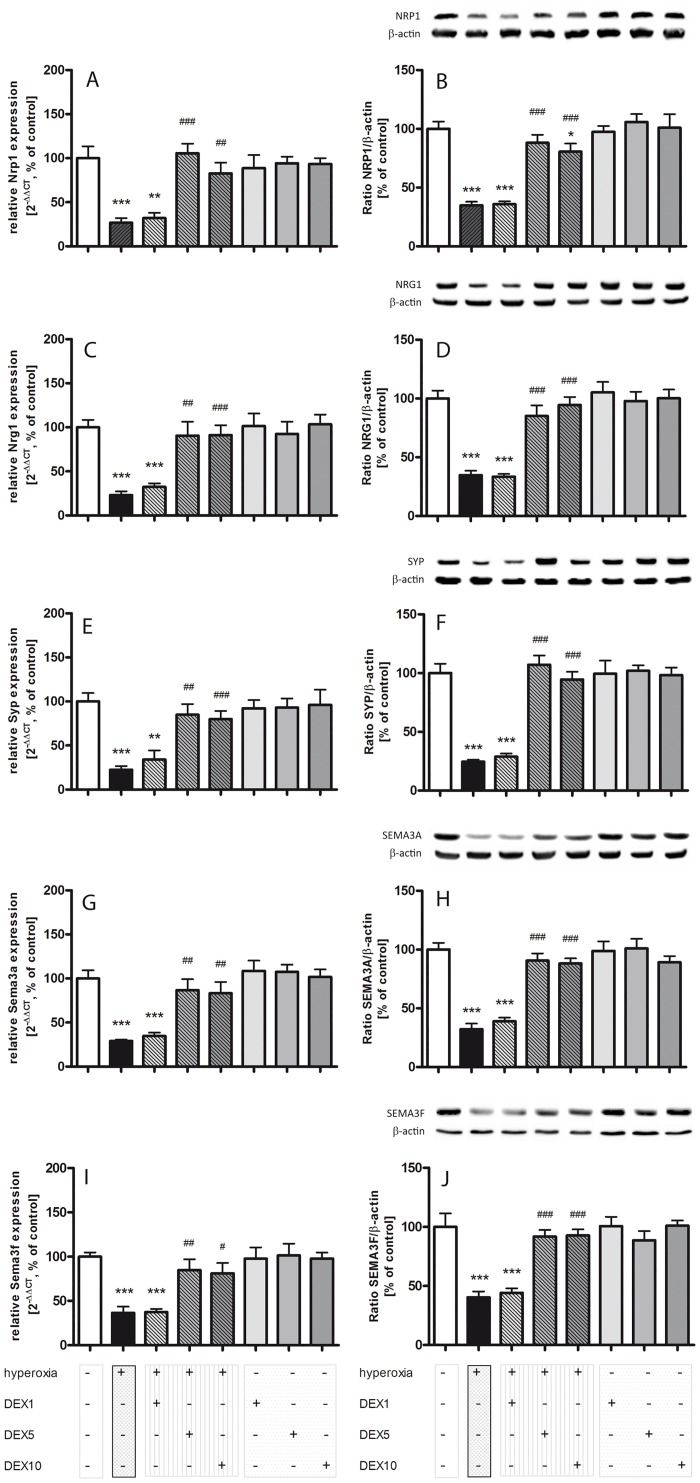
High oxygen concentration resulted in drastically reduced mRNA und protein expression of factors for neurodevelopment and plasticity, and DEX administration reversed these effects. The relative protein and mRNA expression of plasticity factors (Nrp1/NRP1, Nrg1/NRG1, Syp/SYP, Sema3a/SEMA3A, and Sema3f/SEMA3F) were measured in brain homogenates with DEX application (1, 5, and 10 μg/kg) under hyperoxia (hatched grey bars), hyperoxia alone (black bars), DEX under normoxic conditions (plain grey bars), and in comparison to the normoxia control group (100%, white bars) by quantitative realtime PCR and Western blot. We detected in all plasticity factors a significant decrease under hyperoxic conditions for **A-E)** mRNA expression and **F-J)** protein expression. The application of DEX5 and DEX10 under hyperoxia resulted in a significant increase of expression. No changes were measured under normoxia with DEX. Data shown as mean ± SEM, n = 5 per group. ** p<0.01, and *** p<0.001 versus control; ^#^ p<0.05, ^##^ p<0.01 and ^###^ p<0.001 versus hyperoxia (t-test, Bonferroni post hoc test after one-way ANOVA).

Brain development is characterized by a succession of stages which are mediated through neurogenesis, the processing of neuronal migration, maturation, synaptogenesis, and myelination. A variety of internal and external factors can influence and regulate this process before the neurons reach their correct site of action (reviewed in [53]). In this study, we examined various plasticity factors on the mRNA level, which was confirmed at the protein level without exception (see Table 2). The transmembrane and adhesions protein neuropilin 1 (NRP1) can modulate the migration of neuronal progenitor cells [54,55]. Exposure to hyperoxia reduced neuropilin 1 (Fig 4A and 4B) expression widely and was not improved by the administration of DEX1. DEX5 and DEX10, however, significantly reversed this downregulation. NRP1 can also act as the ligand binding subunit of the class 3 semaphorin proteins in neurons [55], with SEMA3A and SEMA3F being the best studied semaphorins [56]. Exposure to hyperoxia resulted in the reduced expressions of both semaphorin 3a (Fig 4G and 4H) and 3f (Fig 4I and 4J), which were increased by a single dose of DEX5 and DEX10. DEX1 revealed no protective effect. Synaptophysin (SYP) is an integral membrane and pre-synaptic protein that is widely used as a marker for synaptic density [57]. The negative effects of hyperoxia were also demonstrated in SYP expression (Fig 4E and 4F). Again, only DEX5 and DEX10 had a protective effect, while DEX1 had no effect on synaptophysin expression. Neuregulin 1 (NRG1) is a cell-cell signaling protein involved in various stages of neural development [58,59] that showed similar expression patterns (Fig 4C and 4D). Hyperoxia reduced the NRG1 expression, DEX5 and DEX10 prevented this reduction while DEX1 had no effect. No influence of DEX was observed on the expression plasticity factors under normoxia.

**Table 2:** Expression profile of plasticity factors in the acute hyperoxia model with dexmedetomidine.

hyperoxia	-	+	+	+	+		-	+	+	+	+
dexmedetomidine	-	-	1 μg/kg	5 μg/kg	10 μg/kg		-	-	1 μg/kg	5 μg/kg	10 μg/kg
*mRNA*						protein					
***Nrp1***	100±13.3	^b^27±5.3	^a^32±6.0	^e^106±10.7	^d^83±12.1	**NRP1**	100±6.1	^b^35±3.1	^b^36±2.5	^e^88±6.8	^e^81±6.9
***Nrg1***	100±8.4	^b^23±4.2	^b^32±4.0	^d^91±15.6	^e^91±11.1	**NRG1**	100±6.6	^b^35±3.7	^b^33±2.4	^e^85±9.1	^e^94±6.9
***Syp***	100±9.6	^b^23±3.9	^a^34±10.5	^d^85±12.2	^e^80±9.3	**SYP**	100±8.0	^b^25±1.8	^b^29±2.9	^e^107±7.8	^e^95±6.5
***Sema3a***	100±9.2	^b^29±1.5	^b^35±3.8	^d^87±12.6	^d^83±12.6	**SEMA3A**	100±5.7	^b^32±4.7	^b^39±3.2	^e^91±6.1	^e^88±4.3
***Sema3f***	100±4.7	^b^37±7.0	^b^37±3.7	^d^85±12.3	^c^81±11.6	**SEMA3F**	100±11.5	^b^40±4.9	^b^44±3.6	^e^92±5.6	^e^93±5.1

Data are expressed as % of control (CON) as mean ± SEM for mRNA and protein expression. n = 5/group, analyzed in identical animals.

^**a**^ p < 0.01, and ^**b**^ p < 0.001 vs. normoxia; and ^**c**^ p < 0.05, ^**d**^ p < 0.01, ^**e**^ p < 0.001 vs. hyperoxia (Bonferroni *post-hoc* test after one-way ANOVA).

***Nrg1*/NRG1:** neuregulin 1; ***Nrp1*/NRP1:** neuropilin 1; ***Sema3a*/SEMA3A:** semaphorin 3A; ***Sema3f*/SEMA3F:** semaphorin 3F; ***Syp*/SYP:** synaptophysin.

### Hyperoxia affects proliferation, maturation and plasticity

**Fig 5:**
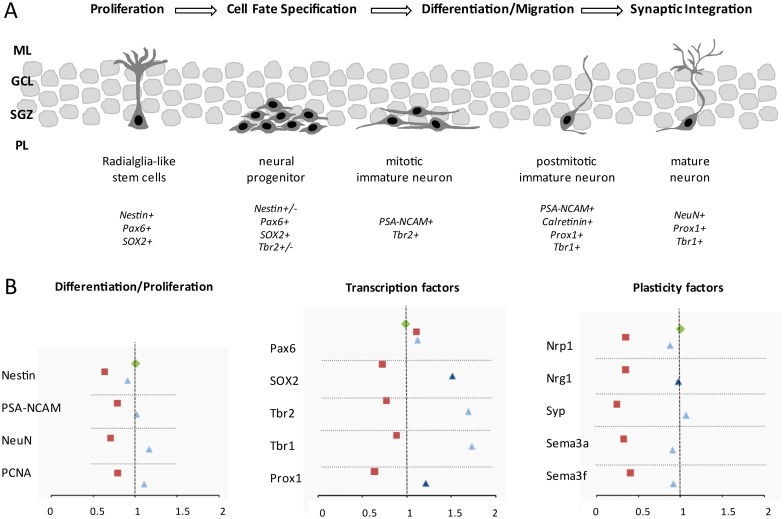
Generation of new hippocampal neurons in the developing brain and modulation with hyperoxia and dexmedetomidine. **A**) Radialglia-like stem cells undergo different stages of processing with proliferation and generation of neural progenitors that can further differentiate into mitotic and postmitotic neurons and finally into mature neurons. These well-orchestrated processes are modulated through different intrinsic factors and it also characterized something like transcription factors and neuronal markers. **B**) The results of our experimental animal study demonstrated impressively the downregulation of neurogenesis- and plasticity-related factors by acute hyperoxia (red square) of the six-day old rat pups compared to normoxia exposure (green rhombus). The α2 agonist dexmedetomidine at concentrations of 5 μg/kg (light blue triangle) and 10 μg/kg (blue triangle) was significantly protective against oxidative stress in the process of neurogenesis and neuroplasticity. **GCL**, granule cell layer; **ML**, molecular layer; **NeuN**, neuronal nuclei; **Nrg1**, neuregulin 1; **Nrp1**, neuropilin 1; **Pax6**, paired box 6; **PCNA**, proliferating cell nuclear antigen; **PL**, polymorphic layer; **Prox1**, prospero homeobox 1; **PSA-NCAM**, polysialylated neuronal cell adhesion molecule; **Sema3a/f**, semaphorin3a/f; **SOX2**, sex determining region Y-box 2; **SGZ**, subgranular zone; **Syp**, synaptophysin; **Tbr1/2**, T-box brain gene 1/2.

Neurogenesis is processed in sequential, overlapping stages (Fig 5A): proliferation, fate specification, neuronal differentiation and migration, and synaptic integration [60]. The neuronal progression is controlled by specific transcription factors [61] and characterized by the expression of neuronal markers [47].

Exposure to high concentrations of oxygen negatively affected the gene expression of these relevant neuronal mediators and proteins as well as essential factors of neuronal plasticity (Fig 5B). The combined results show a significant negative regulation on the expression of neuronal markers and cellular mediators of transcriptional network and neuronal plasticity after hyperoxic exposure compared to normoxic conditions. Dexmedetomidine improved these impairments substantially and thus, DEX appears to be an adequate sedative with neuroprotective effects during the phase of rapid brain growth.

## Discussion

The present study demonstrates the neuroprotective properties of the α2-adrenoreceptor agonist, dexmedetomidine, on neurogenesis and neuronal plasticity, as measured by differentiation and proliferation of neuronal precursors in the dentate gyrus, in a rat model of neonatal oxidative stress induced brain injury.

Neural stem cells (NSC) NSCs are the origin of neurons and glia during embryogenesis and adult neurogenesis throughout life with the plasticity to give rise to new neurons, astrocytes, and oligodendrocytes [44,62–64]. The developmental stages of neurons are well orchestrated through a multistep process and characterized by specific neuronal markers and transcription factors (see Fig 5A). Neuronal progenitor cells are born with a high proliferative capacity for self-renewal and generation of new migrating neurons [47,48], and the developing rat brain, around postnatal day six, mirror those dynamic process of human neurogenesis [62].

The neurotoxic effects of high oxygen concentrations on the development and survival of neuronal cells in the immature rat brain has already been demonstrated in previous studies [12,15,33]. We found a drastic reduction of proliferation capacity in the dentate gyrus of 6-day old rats exposed to high oxygen but not reduction in the number of DAPI positive cells [15,33].

Furthermore, oxidative stress resulted in a strong reduction of the expression of the neural progenitor marker, nestin [49], of PSA-NCAM, expressed in mitotically-maturing neurons [50], and NeuN, a marker for postmitotic mature neurons [51], pointing to a severe delay in hippocampal neuronal maturation. Changes in the expression of proteins involved in neuronal migration, axon growth and guidance after oxidative stress are well documented by Kaindl and colleagues [65]. The oxgen-mediated impairment of neuronal proliferation and maturation was largely inhibited by a single administration of DEX at low or intermediate concentrations (1 and 5 μg/kg). In contrast, high-dose DEX (10 μg/kg) did not enhance proliferation (PCNA+) or differentiation (PSA-NCAM+, NeuN+) after hyperoxic exposure. High-dose DEX appeared to be toxic for mature (NeuN+) neurons in room air, while being protective for nestin-positive progenitor cells under hyperoxic conditions. Nestin marks a niche of stem/progenitor cells with the capacity for proliferation and differentiation [66], being expressed in cells with early neural crest lineage that can differentiate into neurons or glia cells [67]. Thus, high-dose DEX appears to be protective in immature cells (Nestin+) but not in intermediate (PSA-NCAM+) and mature neurons (NeuN+).

Following various CNS injuries, a subpopulation of reactive astrocytes express several stem cell-associated proteins, such as SOX2 and nestin [68]. SOX2 is thought to be critical for NSCs proliferation and differentiation and SOX2-positive cells generate a subpopulation of undifferentiated, dividing cells in the subgranular zone of adult dentate gyrus with multipotent properties [69]. The administration of DEX (10 μg/kg) under hyperoxia resulted in highly increased RNA expression of the transcription factor SOX2, which could contradict the hypothesis that only neuronal differentiation is affected by high dexmedetomidine concentrations. This is accompanied by the observation that at this concentration no protective effect was observed for the intermediate (PSA-NCAM+) and mature neurons (NeuN+).

The development of granule cells in the hippocampus are controlled and regulated by specific transcription factors at different stages of neuronal differentiation [62]. The sequential overlapping expression of transcription factors Pax6, SOX2, Tbr2, Tbr1, and Prox1 correlates with the stage-specific expression of differentiation neuronal markers nestin, PSA-NCAM, and NeuN [52]. It appears that Pax6 and Tbr1, transcription factors from radialglia-like/progenitor cells and the immature postmitotic neurons, were not influenced by hyperoxia. However, SOX2 and Tbr2, transcription factors from undiffentiated and mitotic immature neurons, as well as Prox1, a transcription factor from mature neurons, were reduced under hyperoxia. The delayed neuronal maturation is probably mediated by the reduced expression of these transcription factors [52]. Similar to the neuronal markers, treatment with DEX in hyperoxia-exposed rats rescued the expression of the transcription factors studied.

Levels of neurogenesis can be modulated by various factors, such as environmental enrichment as a positive regulator or diversity of stress as a negative regulator [15,70]. The essential neurogenic zone is located in the region below the granule cell layer in the dentate gyrus of the hippocampus, constituting a life-long supply of new neural progenitor cells that differentiate to mature neurons and glia [47]. These cells, both morphologically and physiologically similar, migrate to the granule cell layer and are functionally integrated with the resident neurons in the existing networks [71]. The newly formed progenitor cells have a high plasticity potential and may switch between a glial and neuronal phenotype [72,73].

In addition to changes in neuronal markers and transcription factors, our experimental hyperoxic conditions significantly altered the investigated plasticity-associated genes expression at both the RNA and protein levels. Specifically, rat pups that experienced high oxygen exposure expressed significantly less Nrg1, a pleiotropic growth and differentiation factor [58], class III semaphorins a/f, with essential functions in patterning of neuronal projections [74], NRP1, a receptor of semaphorin 3a [75], and SYP, a pre-synaptic membrane protein essential for neurotransmission in hippocampal neurons [76], compared to normoxic conditions. In fact, administration of DEX (5 μg/kg and 10 μg/kg) rescued expression of plasticity-associated genes in hyperoxic animals while DEX treatment in controls had no effect. Few changes in overall brain structure or function have been exhibited in Syp knockout mice and major changes in the regulation of neurotransmitter release have been displayed in synaptogyrin/Syp double knockout mice. In particular, short-term and long-term synaptic plasticity was impaired [77]. In a rat traumatic brain injury model, associated with delayed neuronal dysfunction, expression of synaptic proteins was downregulated and reserved by resveratrol treatment. Feng *et al*. postulated that this protection could be associated with the up-regulation of Syp and the suppression of neural autophagy [78]. An oxidative stress-associated diabetic model showed a reduced Syp protein level [79] and this deficiency in Syp induced a decrease in synaptic vesicle, which interfered with the release of neurotransmitters and the synaptic network [80]. Neuroregulins acted as ligands for the epidermal growth factor receptors [81]. An activation of these receptors by NRG1 led to regulation of cell proliferation, migration, and differentiation in different neural systems and promotion of neuron-primed elongation of radialglia and neuronal migration during development [81]. NRG1 deficiency led to disrupted hippocampal plasticity and imbalanced excitatory and inhibitory neurotransmission [82]. Agarwal *et al*. proposed that a balanced NRG1 level is required for synaptic neurotransmission [83]. *In vitro* studies have shown that recombinant NRG1 stimulated neurite outgrowth in primary neurons [84] and neural cell lines [85], indicating that NRG1 is neurotrophic and neuroprotective [86]. Semaphorins are linked to different cellular processes, including proliferation and migration [87], and implicated in synaptic and structural plasticity, neurotransmission, and neurological diseases [88–90]. NRP1 is represented as receptor of SEMA3A and presented in all neuronal populations known to respond to SEMA3A [75]. Hippocampal accumulation of SEMA3A in early stages of Alzheimer´s disease suggested a link to neurodegenerative processes [91].

Very preterm infants have an increased risk for developmental problems including cognitive deficits and behavior disorders [21] that may reflect altered neuronal plasticity. It is known from age-related studies that oxidative stress resulted in cognitive decline [92] and can be improved with neurotrophic factors [93]. Thus, it seems likely that neuroprotection with DEX in an oxidative stress model of the neonatal rat is not only due to improvement of neuronal maturation and differentiation, but apparently also to the preservation of neuronal plasticity. In an isoflurane-induced injury model in 7-day old rats, DEX prevented the neurocognitive deficits [94]. Treatment with DEX abolished the intracerebral hemorrhage-induced impairment of short-term and spatial learning memory [95]. Qian *et al*. described an improvement in early postoperative cognitive dysfunction in aged mice with DEX [96]. Tachibana and collegues investigated the long-term neurological consequences of neonatal administration of DEX and showed that DEX preserved hippocampal synaptic plasticity and synaptic transmission later in life [97]. In our previous study, DEX provided against toxic oxygen induced inflammation and cell death and showed anti-inflammatory, anti-apoptotic, and anti-oxidative properties [33].

Hippocampal development displays important differences between male and female subjects (reviewed [98]). Males have also a higher incidence for prematurity-related mortality and, neurodevelopmental disorders. [99]. Smith and colleagues demonstrated increased behavioral impairment in male, as opposed to female rodents, in a neonatal hypoxia–ischemia model [100], while histopathological damage did not differ by sex. Resilience to noxious conditions may be mediated the presence or absence of sex hormones in the developing brain [101,102]. We failed to find any differences between male and female animals in this study, although it is possible that the use of a larger sample size may have revealed some subtle differences.

In summary, DEX demonstrated neuroprotective properties in our oxidative stress injury model in the developing rat brain, preserving postnatal neurogenesis and neuronal plasticity. DEX increased proliferative capacity of mitotic cells, leading to ultimately higher cell counts of mature neurons, without affecting apoptosis rate in control animals. *In vivo* investigations demonstrated that postnatal DEX did not lead to persisting learning deficits, affected the developing brain, or impaired hippocampal synaptic plasticity [97,103]. Nevertheless, sedating DEX concentrations in adult rats have been associated with altered hippocampal synaptic plasticity [104]. Given the high degree of vulnerability of the developing brain, no potentially beneficial drug can be expected to without side effects.

There are several limitations of this study pointing to areas of future investigations. First, the injury model used is a model of acute oxygen toxicity. Preterm children usually require a longer period of supraphysiological oxygen concentrations. Second, we studied primarily the situation of anti-inflammatory and neuronal neurogenesis and the plasticity-promoting effect of DEX immediately after acute oxygen exposure. Continuing studies at later survival time points would be important as well as functional/behavioral measurements and identifications of morphological changes of neurons (3D reconstruction) in terms of plasticity-promoting effect of DEX. Third, the tested concentrations of dexmedetomidine here are based on clinical concentrations that have been used in children (1 μg/kg) [105] or shown to be mediate neuroprotective effects in animal experiments (5 μg/kg, 10 μg/kg) [97,106] but DEX concentrations in blood or brain tissue were not measured.

In ventilated sick preterm infants, the needs for supplemental oxygen and sedative drugs often coincide. In this setting, DEX appears to be a promising candidate that warrants further investigations.

## Conclusion

The role of DEX has been an interesting topic of neonatological and pediatric anesthetic research in the last years. To define the neuroprotective effects of DEX we investigated the implications of DEX in an oxidative stress model of the developing rat brain with a focus on hippocampal neurogenesis as well as neuronal plasticity. In conclusion, high oxygen and thus oxidative stress caused a delayed differentiation and maturation of neuronal progenitors, reduced proliferation capacity, and impaired neuronal plasticity during the vulnerable stage of brain developing. This can be significantly attenuated by pre-treatment with DEX. DEX under atmospheric conditions improved neurogenesis and enhanced neuronal transcription factor expression. Clinical research into the benefits of DEX on brain development and neurodegeneration is warranted and should include long-term neurodevelopmental follow-up while experimental work should attempt to elucidate the underlying mechanisms by which DEX affects neuronal plasticity in detail.

## Supporting information

S1 Table Original immunohistochemical data. (XLSX)

S2 Table Original RT-PCR data. (XLSX)

S3 Table Original Western blot data. (XLSX)
